# Art of prevention: Atopic dermatitis in women and families of color-prevalence, recognition, and prevention

**DOI:** 10.1097/JW9.0000000000000014

**Published:** 2022-03-29

**Authors:** Britney N. Wilson, Andrew Alexis, Jenny E. Murase

**Affiliations:** a School of Medicine, Rutgers New Jersey Medical School, Newark, New Jersey; b Department of Dermatology, Weill Cornell Medical College, New York, New York; c Department of Dermatology, University of California, San Francisco, San Francisco, California; d Department of Dermatology, Palo Alto Foundation Medical Group, Mountain View, California

**Keywords:** Atopic dermatitis, eczema, skin of color

## Abstract

Atopic dermatitis (AD) is one of the most common skin conditions encountered by dermatologists. Skin of color (SOC) patients, in particular, are 50% more likely to visit a dermatologist for AD than non-SOC patients. While the misdiagnosis of AD in SOC patients is rare, the misinterpretation of severity or undertreatment of disease experienced by this patient population is a common occurrence. Herein, we present this Art of Prevention piece focused on the epidemiology, presentation, treatment, and management of AD in skin of color patients.

**What is known about this subject in regard to women and their families?**

- Atopic dermatitis (AD) causes emotional distress that impairs the quality of life (QoL) of both the children and their parents.
- Mothers made up >85% of prior study populations assessing the impact of AD on QoL.
- Children of Black and Hispanic parents present with more persistent and poorly controlled AD. This further impacts the QoL of the families caring for these patients.

**What is new from this article as messages for women and their families?**

- This article examines the epidemiology of AD, as it relates to skin of color—a disease with great burden in families of color.
- The clinical insights featured in this article have the propensity to improve the quality of care of all patients of color presenting with AD.

## Introduction

Atopic dermatitis (AD), the most common chronic inflammatory skin disease, is a relapsing disorder characterized by pruritus and eczematous lesions, which impairs the quality of life of the mothers taking care of children with the condition.^3^ Although this disorder affects people of all ages and ethnicities, it may have a greater burden on populations of African and Asian descent.^7^ African American (AA), Latino, and Asian patients are 50% more likely to visit a doctor for AD than White patients.^13^ AA were 1.67 times more likely than their White counterparts to be diagnosed with AD.^16^ Several other population-based studies suggest an increase in the burden of AD among Black or Asian children.^5^ Furthermore, Black and Hispanic children are more likely than White children to present with persistent and poorly controlled AD.^1,9^ Factors contributing to the epidemiology of AD are multifactorial. The diverse clinical presentations of AD in darker pigmented skin types can result in misdiagnosis or underrecognition.

## Role of race, ethnicity, and genetics in disease heterogeneity

Differences in race, ethnicity, and genetics may contribute to some of the heterogeneity seen in AD. Filaggrin (*FLG*) null mutations contribute to defects in the skin’s barrier protection and are known to contribute to the development of AD.^20^ Variations in filaggrin loss-of-function (*FLG* LoF) differ significantly by race and their association with the persistence of AD.^12^ The most common *FLG* mutations affect a significantly lower proportion of AA compared with European Americans.^7^ Interestingly, there seems to be a higher prevalence of these mutations in AA with both AD and ichthyosis vulgaris.^26^ In both AA and European Americans, severe AD is associated with small intragenic copy number variation.^27^ Compared with AA children, White children were 2.44 times more likely to carry any *FLG* LoF variant.^12^ Furthermore, there were variants of *FLG* LoF mutation that were only found in children of certain ancestry. For example, *FLG* LoF variants p.S3316 and p.R826 were only seen in AA patients and certain *FLG2* LoF mutations were associated with the persistent AD in AA children.^11,12^ Uncommon fliaggrin variants have been associated with persistent AD in AA.^22^

There are also differences in the immunophenotypic presentation of AD in patients of color. East Asian patients with AD have been shown to have an increased T_H_17 axis compared with European American patients with AD.^9^ However, the T_H_17 axis is attenuated in some AA patients with AD.^15^ There are also racial differences in stratum corneum characteristics that may also contribute to disease severity.^6^ Patients of African origin have the lowest ceramide to cholesterol ratio, whereas Asian patients have the highest ratio.^6^ These findings may be relevant to observed clinical differences among patients with AD.^6^

## Varying clinical presentations

**Fig. 1.**
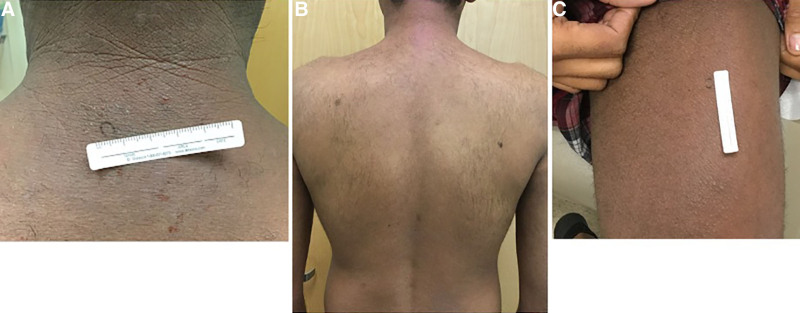
Full-body erythroderma masked by the darker pigmentation of this patient’s skin.

These aforementioned immunophenotypic and genetic variations along with other factors may contribute to the different clinical presentation seen in skin of color patients with AD. In richly pigmented skin, erythema may appear less red and take on a dark brown, or purplish hue with elements of gray scaling.^7,17^ Figure 1A–C reveals a patient who had full-body erythroderma, but the visibility of erythema is altered by background pigment.

In addition to differences in hue, there are also differences in the morphology and secondary characteristics in skin of color patients with AD. In Asian patients, AD commonly presents as well-demarcated prominent scaling and lichenification with some features reminiscent of psoriasis.^14^ The follicular variant of AD, which presents as 1–2 mm perifollicular accentuation and papules on the extensors and trunk is more common in patients with darker skin types.^7^ AD can also present as papular lichenoid lesions and papular nodules that occasionally coalesce into thick hyperchromic plaques in patients of African and Asian descent.^7^ Patients of African origin are more likely to develop prurigo nodularis, periorbital dark circles, and lichenification in the setting of AD.^7^ This is believed to develop due to greater pruritus and consequential increased rubbing and scratching.^7^ These differences in clinical presentation may contribute to the numbers of per capita visits cited by Janumpally et al^5^ whose research revealed the health care utilization for AD in Black and Asian/Pacific Islander patients is 2-fold and 6-fold higher, respectively, than their White counterparts.

## Practical interventions: Look, listen, feel

**Fig. 2.**
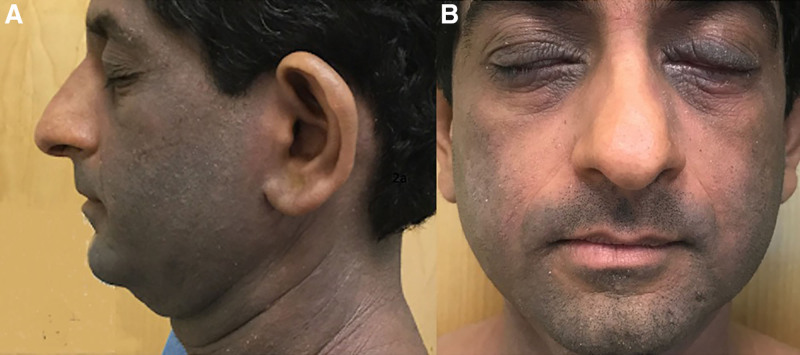
Sebaceous areas around nose revealing baseline skin color.

To avoid underestimating the severity of AD or the presence of erythroderma, close inspection of islands of nonlesional skin, side-lighting, and palpation may help. In Figure 2, it becomes apparent that the sebaceous areas around the nose and nasolabial folds show the patient’s baseline skin color, but the lateral cheeks demonstrate the erythroderma. The density of sebaceous glands in this location helps to prevent the masking of erythema. In Black children, it is imperative that dermatologists avoid reliance on the classical presentation of erythema in diagnosing AD as reliance on erythema scores (as measured by perceived redness) may lead to the underdiagnosis and undertreatment of severe AD.^28,29^ Palpation may also allow for better appreciation of the scale, papulation, and induration of active lesions. As erythema may be more subtle in richly pigmented skin types, symptomatology including pruritus is particularly helpful in identifying active AD lesions in a given patient. For severity assessments, we encourage the use of validated pruritus instruments for a more accurate assessment such as ItchyQol.^4^ Questions like, “Does the itching distract activities during day?” and “Does the itching wake you up at night?” may be helpful in gauging the degree of pruritus. It can be helpful to request photos from the patient, for example, on the patient’s mobile photo or driver’s license, when their AD was under better control as a point of comparison. In addition, asking the patient’s perspective of the degree of erythema (“Is this red for you?”) is also beneficial since the patient knows their baseline skin tone and can provide feedback to the provider regarding the degree of inflammation. Inviting your patient’s input is an essential component to implementing active communication skills that foster the development of a healthy patient-doctor relationship.^19^ Herein, we present practical interventions that can be used when diagnosing skin of color patients with AD based upon prior successful clinical experiences and a review of the literature.

## Conclusions

There is a complex interplay between genetics, immunology, environment, and other unknowns that may influence the clinical presentation of AD in patients of color. It is critical that dermatologists are aware of the various presentations and different morphologies of AD. It is our hope that the clinical pearls and knowledge shared in this article will help clinicians in diagnosing and treating patients of all races and ethnicities suffering from AD.

**Table 1.** Summary of key clinical insights regarding managing atopic dermatitis in skin of color patients

Avoid underestimating the severity of atopic dermatisis in SOC patients by using close inspection of nonlesional skin, side-lighting, and palpation.
Acknowledge the degree of erythroderma by closely observing highly sebaceous areas like the nasolabial fold, which are more likely to reveal SOC patients’ true color.
Palpation allows for better appreciation of the scale, papulation, and induration of active lesions.
Use a validated pruritus instruments like ItchyQol for a more accurate assessment of severity.

## Acknowledgments

We would like to acknowledge the patient from whom we obtained informed consent to participate in this publication.

## Conflicts of interest

None.

## Funding

None.

## Study approval

N/A.

## Patient consent

Informed, written consent was received from the patient and confirmed to the journal prepublication, stating that the patient gave consent for their photos and case history to be published.
